# Web-Based Training for Nurses on Shared Decision Making and Prenatal Screening for Down Syndrome: Protocol for a Randomized Controlled Trial

**DOI:** 10.2196/17878

**Published:** 2020-10-29

**Authors:** Alex Poulin Herron, Titilayo Tatiana Agbadje, Melissa Cote, Codjo Djignefa Djade, Geneviève Roch, Francois Rousseau, France Légaré

**Affiliations:** 1 Canada Research Chair in Shared Decision Making and Knowledge Translation Université Laval Québec, QC Canada; 2 Centre de recherche en santé durable (VITAM) Québec, QC Canada; 3 Faculty of Nursing Université Laval Québec, QC Canada; 4 Centre Intégré Universitaire de Santé et Services Sociaux de la Capitale-Nationale Québec, QC Canada; 5 Centre Hospitalier Universitaire de Québec - Université Laval Research Centre Hôpital Saint-François d'Assise Québec City, QC Canada; 6 Department of Molecular Biology Medical Biochemistry and Pathology Université Laval Québec City, QC Canada; 7 Department of Family Medicine and Emergency Medicine Université Laval Québec, QC Canada

**Keywords:** shared decision making, prenatal screening, training, nurses, behavioral intention, Down syndrome, continuing professional development

## Abstract

**Background:**

Pregnant women often find it difficult to choose from among the wide variety of available prenatal screening options. To help pregnant women and their partners make informed decisions based on their values, needs, and preferences, a decision aid and a web-based shared decision making (SDM) training program for health professionals have been developed. In Canada, nurses provide maternity care and thus can train as decision coaches for prenatal screening. However, there is a knowledge gap about the effectiveness of SDM interventions in maternity care in nursing practice.

**Objective:**

This study aims to assess the impact of an SDM training program on nurses’ intentions to use a decision aid for prenatal screening and on their knowledge and to assess their overall impressions of the training.

**Methods:**

This is a 2-arm parallel randomized trial. French-speaking nurses working with pregnant women in the province of Quebec were recruited online by a private survey firm. They were randomly allocated (1:1 ratio) to either an experimental group, which completed a web-based SDM training program that included prenatal screening, or a control group, which completed a web-based training program focusing on prenatal screening alone. The experimental intervention consisted of a 3-hour web-based training hosted on the Université Laval platform with 4 modules: (1) SDM; (2) Down syndrome prenatal screening; (3) decision aids; and (4) communication between health care professionals and the patient. For the control group, the topic of SDM in Module 1 was replaced with “Context and history of prenatal screening,” and the topic of decision aids in Module 3 was replaced with “Consent in prenatal screening.” Participants completed a self-administered sociodemographic questionnaire with close-ended questions. We also assessed the participants' (1) intention to use a decision aid in prenatal screening clinical practice, (2) knowledge, (3) satisfaction with the training, (4) acceptability, and (5) perceived usefulness of the training. The randomization was done using a predetermined sequence and included 40 nurses. Participants and researchers were blinded. Intention to use a decision aid will be assessed by a t test. Bivariate and multivariate analysis will be performed to assess knowledge and overall impressions of the training.

**Results:**

This study was funded in 2017 and approved by Genome Canada. Data were collected from September 2019 to late January 2020. This paper was initially submitted before data analysis began. Results are expected to be published in winter 2020.

**Conclusions:**

Study results will inform us on the impact of an SDM training program on nurses’ intention to use and knowledge of decision aids for prenatal screening and their overall impressions of the training. Participant feedback will also inform an upgrade of the program, if needed.

**Trial Registration:**

ClinicalTrials.gov NCT04162288; https://clinicaltrials.gov/ct2/show/NCT04162288

**International Registered Report Identifier (IRRID):**

DERR1-10.2196/17878

## Introduction

Choosing whether to undergo prenatal screening is a difficult decision for pregnant women, and they are rarely prepared for or supported in that decision [1]. Shared decision making (SDM) fosters decisions that reflect the best available evidence and what matters most to patients [2]. Evidence suggests that SDM is the best practice for informed consent [3]. SDM is now part of policy and legislation in many countries for ethical, social, and economic reasons [4]. According to the literature, SDM appears to improve patients’ and clinicians’ health care experiences, health care processes, patient outcomes, and health costs [5]. SDM also seems to reduce the overuse of ineffective tests and treatments and increase the uptake of effective ones [6]. It could thus play an important role in reducing harms and increasing patient safety [7]*.* Patient decision aids (DAs) are SDM tools that foster the involvement of patients in decisions by specifying a decision point, informing them of options and outcomes, and helping them clarify what matters most to them [8,9].

Nevertheless, SDM is rarely implemented in prenatal care [10]. Pregnant women are rarely given a chance to weigh the advantages and disadvantages of undergoing prenatal screening or to identify what matters most to them [1]. This can translate into discomfort with decisions (decisional conflict), decision regret, and potential complaints [11-13]. Results of systematic reviews indicate that SDM would be implemented if clinicians and patients had access to DAs, if providers were trained in SDM, and if public awareness campaigns about SDM were carried out [8,14]. However, despite an increase of 174% in SDM training programs in 4 years (2011-2015), only about 29% of these programs were evaluated [15]. Thus, there is little known about their overall effectiveness [15].

The province of Quebec, in Canada, offers each pregnant woman the opportunity to screen for Down syndrome with the serum-integrated prenatal screening test (which includes nuchal translucency) [16]. Currently, noninvasive prenatal testing is only offered in private institutions. Since several prenatal screening tests are available, health care professionals must be well-informed about the risks and probabilities surrounding screening results and must be able to communicate these to pregnant women in their care. Thus, effective informational resources, tools, and training are urgently required.

The members of the Canada Research Chair in Shared Decision Making and Knowledge developed a training program to support health care professionals in practicing SDM in the context of prenatal screening. The program was developed with the help of 5 professionals (family medicine doctors, biochemical doctors, ethicists, and scientists) who provided expertise on SDM, prenatal screening, and ethics. Their expertise is conveyed through videos in which experts respond to questions related to each module. Although the Research Chair has developed some SDM training programs [17,18], this program for prenatal screening is new; and no training evaluation, such as focus groups or usability testing, have been undertaken.

Nurses can play a larger role with pregnant women in prenatal screening. Those not already doing so can provide information and counseling about prenatal screening [19] and implement and evaluate it [20]. Patients themselves have suggested that nurses could provide significant help in SDM. Nurses already explain relevant medical notions, support the patients, and communicate with other clinicians [21]. However, to engage in SDM with future parents, nurses must be aware of evidence-based information on the kinds of screening available and must take the future parents’ preferences into consideration. SDM training could be a way to implement this approach in nursing practice. While most SDM implementation studies focus on physicians [13], health care reforms are resulting in nurses taking more responsibilities [22], and their role in SDM will likely increase. It is therefore timely to address the gap in the literature on the effectiveness of SDM training, especially for nurses and for prenatal screening.

The primary objective of this study is to assess the impact of an SDM training program on the intention of nurses to use a DA to support prenatal screening decisions among pregnant women. The secondary objectives are to assess the impact of the training on knowledge related to SDM and prenatal screening as well as to assess nurses’ overall impressions (satisfaction, acceptability of the training, and perceived usefulness) regarding the training. It is expected that this web-based training program will significantly increase nurses’ intention to use a DA and will increase their knowledge about SDM and prenatal screening. It is also hypothesized that nurses will perceive this training as relevant and useful.

## Methods

### Study Design

This study is a 2-arm randomized controlled trial. Participants were randomly allocated to 2 parallel groups: (1) an experimental group exposed to a 3-hour web-based training program on SDM, including SDM for prenatal screening (n=18), or (2) a control group exposed to a 3-hour web-based training program on prenatal screening alone (n=18). The CONSORT-EHEALTH (Consolidated Standards of Reporting Trials of Electronic and Mobile Health Applications and Online Telehealth) checklist (V.1.6.1) will be used as a reporting guideline [23].

### Research Approval

This project was approved by the ethics committee of the Centre Hospitalier Universitaire de Québec-Université Laval (MP-20-2019-4571). All stages of this research project will be carried out in accordance with the rules of ethics. If any amendment to the protocol is required, it will be submitted to the ethics committee and stated in the final paper. All participants consented to their participation in the research project before starting the study. The consent form stated that the participants had the right to refuse to participate and the right to withdraw at any time without providing any justification and without prejudice to preexisting entitlements.

### Study Population

Inclusion criteria for nurses included those who (1) supported prenatal screening decision making or were involved in prenatal screening processes in the province of Quebec, (2) spoke and wrote French, (3) were active in professional practice during the year of data collection (eg, hospitals, community clinics), and (4) had enough internet skills (all procedures except recruitment were web-based, requiring a minimum of ability and equipment to enter and navigate through the web-based training program). There were no exclusion criteria.

### Procedures and Recruitment

Participants were recruited online by a private polling firm that operates an internet panel. Members of this panel are nurses working in different areas and specialties, and they were invited by email to participate in the study. The polling firm also posted advertisements on social media to attract a larger number of nurses and sent emails to the human resources departments of 2 regional health authorities, the CIUSSS (Centre intégré universitaire de santé et services sociaux) of Chaudière-Appalaches and the CIUSSS of the Capitale-Nationale, asking them to share the study details with their employees. All 3 recruiting methods informed potential participants of how to contact the polling firm recruitment team.

**Figure 1:**
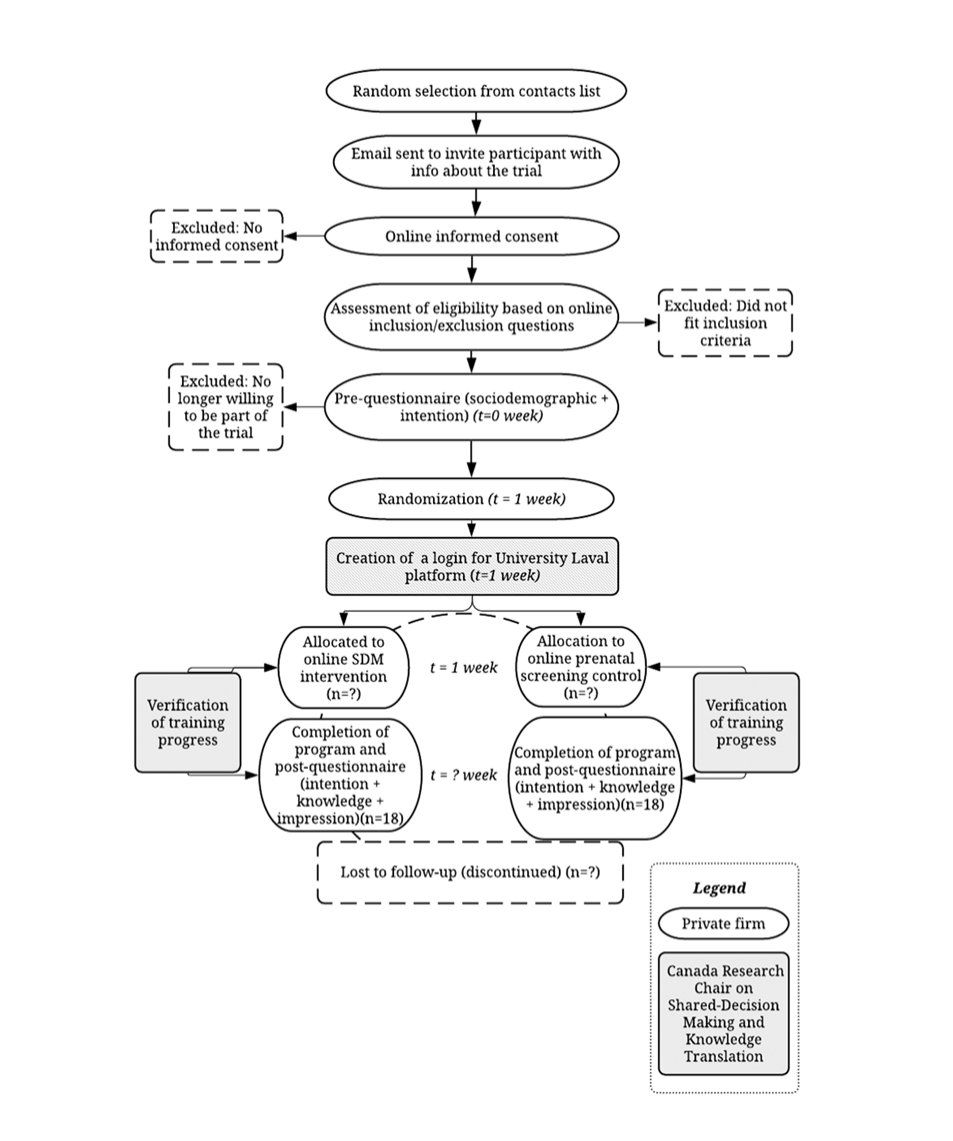
Participant timeline. SDM: shared decision making.

If a participant contacted the firm to express interest in participating in the study, a member of the firm’s recruitment team verified the participant's eligibility by asking some questions. Once the participant's eligibility was confirmed, the polling firm sent the participant the consent form in a first email. After receiving the consent forms, the private polling firm then sent a second email with a link to the preintervention questionnaire. The preintervention and postintervention questionnaires were programmed on the polling firm’s platform, and a hyperlink to access them was inserted in the emails to be sent to the participants. Completion of the preintervention questionnaire was a prerequisite for accessing the training. Once the preintervention questionnaire was completed, the participant was randomly assigned to the intervention or control group. A login name and password for accessing the Université Laval training platform were then generated for each participant (unless they already had one). All information required to access the training and the link to the postintervention questionnaire (with instructions to complete once the training was completed) was emailed to participants. Participants were allowed a month to complete the training. After receiving access to the training, participants were asked to work through the modules and answer the quizzes at the end of each module. When the participants completed the training, they could answer the postintervention questionnaire. Weekly follow-ups and reminders to complete the training were sent to the participants if needed. The polling firm maintained a contact with participants via their personal emails. If assistance was needed by participants from either randomized group, they could either email the principal investigator or contact computer services at Université Laval. Figure 1 shows the participant timeline according to the SPIRIT (Standard Protocol Items: Recommendations for Interventional Trials) guidelines [24].

### Randomization

The allocation of participants to trial groups was performed after collecting the sociodemographic data. These data were needed for the creation of the login name on the Université Laval platform, and were therefore, mandatory for accessing the training. For simple randomization, before the study started, the polling firm generated a random allocation sequence by computer, enrolled participants, and assigned them to one or the other of the study groups. Participants were blinded throughout the study. However, participants could find out which intervention was the experimental one and which one was the comparator by reading the informed consent procedures (where the desired training effect was indirectly stated). The videos of experts were recorded beforehand and were delivered asynchronously so that the experts/trainers were blinded to participants. The data analysts will also be blinded with respect to allocation groups until they have completed the analysis. One of the members of the research team was not blinded, as she needed to follow the completion of the training program by participants.

### Study Interventions

Participants had to complete a web-based training program, but the content differed according to the group (control or experimental) to which the participant was randomized. The major differences were the SDM component and SDM-specific materials, which were missing in the training for the control group. In other words, in both arms the participants were exposed to a web-based training program, but only the intervention arm exposed the participants to the SDM component and SDM-specific materials. The training program was designed to adapt to the learning pace of users, who could leave the training and return later. For the purposes of this study, no major changes were made to the program during the evaluation process. Moreover, participants could consult other information sources during their training.

#### Intervention Group: Web-Based Training Program on SDM and Down Syndrome Prenatal Screening

**Figure 2:**
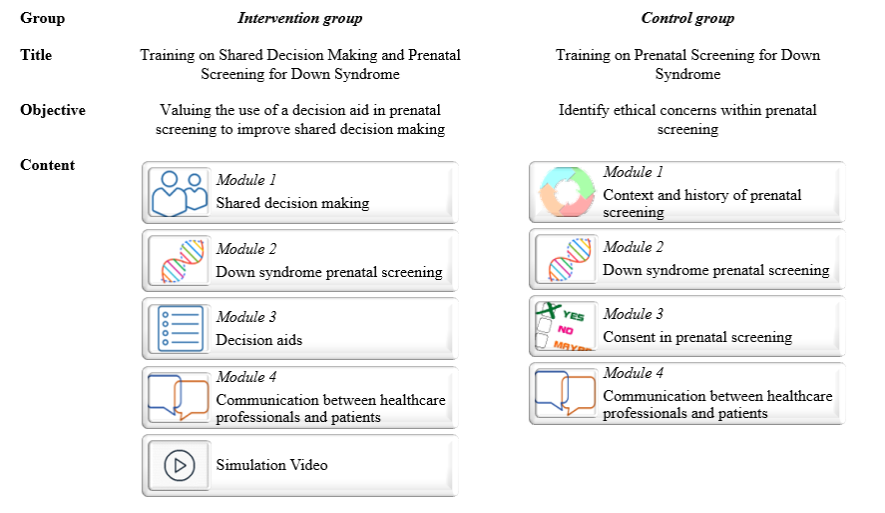
Description of interventions.

The intervention consisted of a web-based self-study training program entitled *Formation en ligne – La prise de décision partagée pour le test de dépistage prénatal de la trisomie 21* (Shared Decision Making About Prenatal Screening for Down Syndrome). This program lasted 3 hours and aimed to integrate SDM into prenatal care. The training program was divided into 4 main modules: (1) shared decision making, (2) Down syndrome prenatal screening, (3) decision aids, and (4) communication between health care professionals and patients (Figure 2). This sequence was chosen to provide an overview of how SDM was defined, to highlight its benefits, to put the approach within the context of prenatal screening, and to provide concrete ways to implement SDM in clinical practice. In each module, the targeted learning objectives were presented along with the work to be carried out (eg, completing readings, watching a video, or filling in an evaluation form). A variety of teaching methods and media were used: videos, interviews, narrated capsules (explanatory videos with verbal explanations), readings, links to scientific articles, and complementary websites. At the end, a simulation video helped learners put the knowledge acquired during training into practice. It was strongly recommended that the users followed the order of presentation of the modules as their sequence was designed to promote progressive learning.

#### Control Group: Web-Based Training Program and Down Syndrome Prenatal Screening

The control group underwent a 3-hour web-based self-study training program but without the SDM component. It was entitled *Formation sur le dépistage prénatal de la trisomie 21* (Training on Prenatal Screening for Down Syndrome). In this program, the topic of SDM in Module 1 was replaced with “Context and history of prenatal screening,” and the topic of DAs in Module 3 was replaced with “Consent in prenatal screening” (Figure 2). This consent module did not include key features of SDM, such as determining the decision points, providing science-based evidence for the pros and cons of options, clarification of patient values and preferences, or the use of a DA. As in the experimental group, each module had target learning objectives. The same teaching methods were used, except that in the 2 control modules, narrated capsules and reports replaced the videos of experts. The simulation at the end was removed because it focused exclusively on SDM and DAs.

### Data Collection

For each study group, 2 data collection periods were planned, before and after the training programs. All outcomes were self-reported. No postintervention data were collected on participants who discontinued the intervention. The Kirkpatrick and Kirkpatrick model [25,26], a rigorous framework for evaluating training, was used as a guide. It divides effectiveness of a training program into 4 levels: (1) reaction to the training, (2) learning due to the training, (3) behavior following the training, and (4) results, such as a reduction in costs or better outcomes for the patient due to the training [25]. Although mid- and long-term outcomes are important for determining behavioral change, for the purposes of this study, these data were not collected.

#### Primary Outcome

The primary outcome was the intention to use a DA in clinical practice after completing the web-based training program on SDM in prenatal screening. The primary outcome was measured preintervention and postintervention (within 24-72 hours of completing the training, as duration is variable).

The intention to use a DA was chosen as an outcome because it facilitates the implementation of SDM in clinical practice [27]. Intentions have already been documented as a strong measure of predicting a behavior [28]. This outcome could predict nurses’ mid- or long-term behavior in clinical practice after receiving the training, that is, it could match the third level of evaluation suggested by the Kirkpatrick and Kirkpatrick model [29].

#### Secondary Outcomes

The secondary objectives were to assess the impact of training on (1) knowledge related to SDM and prenatal screening, and (2) nurses’ overall impression of the training, including satisfaction, acceptability, perceived usefulness, and reaction (to the pedagogical methods). All secondary outcomes were evaluated within 24-72 hours of completing the training.

#### Measures

The Continuing Professional Development Reaction (CPD Reaction) questionnaire [30] was used to measure behavioral intention. CPD Reaction is a validated questionnaire (Cronbach α ranging from .77 to .85) for evaluating continuing professional development, as the name suggests [29]. The 12-item questionnaire scores on 5 constructs: intention, social influence, beliefs about capabilities, moral norm, and beliefs about consequences. This study focuses on intention; however, the other constructs were also evaluated for their potential to predict the behavior of interest.

After receiving the intervention, participants were invited through the postintervention questionnaire to evaluate their knowledge. Knowledge was explored using 20 questions: 2 questions on Down syndrome, 7 on prenatal screening, 7 on SDM, and 4 on ethics. This questionnaire was created by the Canada Research Chair on Shared Decision Making and Knowledge Translation based on advice by an SDM expert (FL), numerous studies of SDM [2,9,31], and governmental information on prenatal screening [16]. Questions were also structured following Bloom’s taxonomy of cognitive learning objectives [32].

Satisfaction was measured regarding the content, trainers, and overall satisfaction using a self-reported questionnaire created by Schmidt [33] and adapted for this study.

The measure of acceptability of the training program was based on a questionnaire by Kasper et al [34]; and questions addressed the comprehensibility, the amount of information, the quality of information, and the chosen format of the training.

The measures of perceived usefulness were based on a questionnaire by Giangreco et al [35]. It considered usefulness in terms of work responsibilities, relevance of topics to career development, relevance of topics in relation to individual learning needs, consistency with declared objectives of the training mentioned at the beginning of each module of both training programs, and balance between theory and practice.

Finally, the measure of nurses’ reaction to the pedagogical aspects of the training used the Kirkpatrick and Kirkpatrick questionnaire, which assesses the general relevance and utility of the training for clinical practice [25].

#### Other Data to be Collected

Participants were invited to complete a sociodemographic questionnaire before accessing the training for two reasons: to have a broad picture of the participants and to extract the information required to create a personal username for the Université Laval web platform through which they were to access the training. At the end of the intervention, participants were asked an open qualitative question about their suggestions for improvement.

### Data Management

All data collected will be kept on the secure server of the polling firm for 10 years. Following data collection, the firm sent a deidentified database of all data collected in an Excel file and a Statistical Analysis Software (SAS) file to the research team. An identification number was assigned to each participant to track them throughout the study. The research team saved these data on the secure server of the CIUSSS-CN (Centre intégré universitaire de santé et services sociaux de la Capitale-Nationale).

### Sample Size

The sample size was determined in reference to a previous study in the field [27] that examined the intention to use a DA for Down syndrome screening among other prenatal care providers, namely gynecologists, general practitioners, and midwives. The mean intention score for midwives in this study was 5.78 (SD 0.84). Midwives’ intention was selected as a point of reference because of their close affinities with nursing practice [36,37]. To detect an average difference between 2 independent groups, it was estimated that a sample size of 36 nurses (n=18 per group) would be enough to detect a difference in intention of using a DA with an error of 0.05, a size effect of 0.8, and power of 80%.

### Data Analysis

Descriptive statistical analysis of sociodemographic characteristics will be performed to ensure the comparability of groups (intervention and control). The *t* test will be performed on the mean of the intention to use the DA in both groups and on knowledge scores. Secondary outcomes (knowledge and overall impression) will be assessed by doing bivariate and multiple regression analyses. For each outcome analyzed, according to the type of variable (continuous or categorical), the degree of fit and the assumptions of each model will be assessed. The statistical significance threshold is a *P* value of <.05, and all statistical analyses will be performed using the SAS statistical package (SAS Institute). No subgroup analysis is planned as of yet.

## Results

This study started in September 2019, and all data were collected by January 2020. Statistical analyses and submission of a paper for publication are anticipated by the end of 2020.

## Discussion

It is expected that this study will provide information about the impact of training on the adoption of SDM skills, such as using a DA, among nurses in prenatal screening. It is expected that this web-based training program will significantly increase nurses’ knowledge about SDM and prenatal screening and will strengthen their intention to use such a tool in their practice.

Regarding strengths of this study, the web-based training was created by a team that has 15 years of experience in the development of SDM tools, including DAs and continuing professional education programs, and has been specifically working on tools for prenatal screening decisions for more than 7 years. This training program was created in collaboration with Université Laval, an institution that can accredit continuing professional development. The randomized controlled trial is a strong study design for evaluating the effectiveness of interventions, as it reduces bias and is a rigorous tool for examining cause-effect relationships between interventions and their outcomes [38]. Participants come from different parts of the province of Quebec, and thus the study will be representative of different types of practice and demographic profiles (eg, rural and urban). Moreover, participants are active health care professionals, and their perspectives will reflect the realities of current practice and their SDM needs.

As for limitations, the first is that our results do not address the fourth level of the Kirkpatrick and Kirkpatrick model (2016). As the program is web-based and focuses exclusively on nurses, outcomes related to women and their partners, such as reduction of decisional regret, could not be examined. In addition, nurses are not the only health care professionals who discuss prenatal screening. The perspective of others should be integrated into the future implementation of this web-based program. Moreover, the sample size does not allow for an examination of the particularities within nursing practice, such as differences between registered nurses and nurse practitioners. Furthermore, the intention of using a DA was only measured once; therefore, it is not possible to know whether this program has a long-term effect on intention. Finally, participants could acquire parallel knowledge in their clinical practice or through curiosity while doing the training, and this knowledge may interfere with results.

Following analysis of the study results, the training program will be improved in line with participants’ contributions. Health care providers’ opinions, in this case the nurses’ perspectives, provide critical input for upgrading training. A training program that nurses consider useful and acceptable is more likely to be adopted by nurses and the institutions in which they work with expecting parents. To date, evaluations of SDM interventions, especially in nursing, are rare. This study will be informative about the effectiveness of such training and can promote implementation of SDM in all health care practices.

## Appendix

Multimedia Appendix 1 Peer review report.

Multimedia Appendix 2 CONSORT-eHEALTH checklist (V 1.6.1).

## Abbreviations

CERSSPL-UL: Centre de recherche sur les soins et services de première ligne de l'Université Laval
CIUSSS: Centre intégré universitaire de santé et services sociaux
CIUSSS-CN: Centre intégré universitaire de santé et services sociaux de la Capitale-Nationale
CONSORT-EHEALTH: Consolidated Standards of Reporting Trials of Electronic and Mobile Health Applications and Online Telehealth
CPD Reaction: Continuing Professional Development Reaction
DA: decision aid
PEGASUS: PErsonalized Genomics for prenatal Abnormalities Screening USing maternal blood
RRISIQ: Réseau de recherche en interventions en sciences infirmières du Québec
SDM: shared decision making
SPIRIT: Standard Protocol Items: Recommendations for Interventional Trials
