# Supplementary material for: Web-Based Training for Nurses on Shared Decision Making and Prenatal Screening for Down Syndrome: Protocol for a Randomized Controlled Trial
Source: JMIR Res Protoc. 2020 Oct 29;9(10):e17878. doi: 10.2196/17878 (PMC7661244; doi:10.2196/17878)
Supplement: Multimedia Appendix 1 [file resprot_v9i10e17878_app1.pdf]

GenomeCanada

2017 Large-Scale Applied Research Project (LSARP) Competition
Genomics and Precision Health

Full Application Review - Summary of Review

Project Leader(s): François Rousseau, Sylvie Langlois

Project Title: PEGASUS-2 - PErsonalized Genomics for prenatal Abnormalities Screening USing maternal blood : Towards First Tier Screening and Beyond

---

A. RESEARCH PROPOSAL, INCLUDING RESEARCH ON (GE^{3}LS)

Strengths:
This is a large-scale clinical trial in an important and contentious area, where evidence of sensitivity/specificity and cost benefits will be very useful. Separating out the different primary and secondary outcome metrics in the trial - all of which are important - is valuable, and evidence to address all of them for T21/T13/T18 screening is likely to be achievable within the proposal. The RTC design is innovative, and will address many of the outstanding questions around the implementation of NIPS as a first-line screening test for fetal aneuploidies and, specifically, what effect removal of the current first-line screening test will have on detection rates of anomalies other than the common trisomies.

The GELS components are integral to the proposal with a focus on implementation. The GELS methods are well designed and fairly clearly detailed in the proposal with enough detail about methods and analysis to understand their goals and approach. The team verified that they intend to draw from previously validated measures for their various quantitative studies regarding measures such as decision conflict and regret, satisfaction, etc.

Weaknesses:
The panel had concerns around the analysis plans for the RCT. Specifically, the description of statistical power calculations and data analysis were either very superficial or technically incorrect. The applicants' responses to questions about these points were unsatisfactory, deferring to members of the team who were absent. The committee strongly recommends that experts in clinical trial design and data analysis are fully engaged in this project.

Although the science in this study is not particularly novel, as it is primarily an implementation trial and GELS study, the panel felt this was not problematic.

Summary Statement:
This is an excellent proposal, which impressed the panel. We have only one minor recommendation for improvement, which is that the study team should carefully evaluate their analytical plan within the RCT to ensure that the correct statistical tests are performed.

B. SOCIAL AND/OR ECONOMIC BENEFITS

Strengths:
This application addresses an important public health area, where potentially large numbers of individuals will benefit. In addition to the benefits outlined in the grant (earlier diagnosis, reduced false positive screens, etc.), moving to first tier NIPS would increase equity of access to fetal anomaly screening across Canada, which is impacted by unequal availability of nuchal translucency screening.

Weaknesses:
Economic benefits are highly dependent upon the ability of the team to reduce the cost of NIPS testing, since the current first-tier screening protocol is significantly cheaper. However, the team are well aware of the trade-offs between cost and test performance, and have taken this into account in their proposal.

There is a risk that next generation low-cost NIPS commercial tests, if developed and deployed on a platform other than NGS, might result in a much lower-cost system (estimated at $50) than that proposed here, and make many of the findings here less relevant, particularly the efforts to reduce price.

By changing Canada's prenatal screening to focus on the common trisomies, and eliminating FTS, it is possible that we will then under-detect the individually rarer, but collectively more common and severe genomic conditions.

Summary Statement:
The potential social and economic benefits of this proposal are substantial, as using NIPS as a first-tier screening test for all pregnant women in Canada could directly affect the service for >450,000 individuals per year, and eliminate ~10,000 false positive screens. The benefits are realisable within or shortly after the timeframe of the grant.

C. MANAGEMENT AND FINANCE

Strengths:
The team has extensive expertise and experience in this area, having successfully delivered on PEGASUS1.

Weaknesses:
None identified.

Summary Statement:
An excellent team whom we believe can deliver their proposal.

GenomeCanada

SUMMARY STATEMENT:

The panel was impressed with the proposal and the presentation by the team. Some minor prior concerns about the proposal were dealt with during the presentation, and the panel was unanimous in its support for this proposal.

GenomeCanada GenomeQuébec

III. PROJECT TEAM

In a maximum of two (2) pages using the table below provide details for key members of the team including end-users. Attach a short CV (maximum of 2 pages) in Appendix II for up to five key team members.

(D=decision maker government/para-gov.; G= Geneticist; L=Clinical laboratory physician; O=Obstetrician; P=Primary care physician; E= Epidemiologist; H = Health Services Researcher; S= ELSI)

Last Name	First Name	Affiliation	Role[2]	Time Commitment (hrs/ week)	Description of Contribution & Reasons for Inclusion in Proposal
Rousseau *L	François	U.Laval	Project Leader Act 1 Lead	20	Project scientific and financial management, Mol Dx, HTA, EBLM
Langlois *DG	Sylvie	UBC	Project Co-leader Act 1 Co-lead	10	Scientific and clinical management, Prenatal screening & guideline expert, UBC site leader
Reinharz *H	Daniel	U.Laval	Act 2 Lead – Co-app.	10	Health economics, public health expert
Ravitsky *S	Vardit	UdMontréal	Act 3 Lead – Co-app.	10	Ethical and social issues expert, NIPD
Légaré *P	France	U. Laval	Act 4 Lead – Co-app.	3	GP, Shared decision making, KT expert, implementation res.
Wilson *H	Brenda	U Ottawa	Co-Lead. Act 4 study design and analysis	2	Public health genetics, KT
Caulfield *S	Timothy	U Alberta	Co-Lead Act 3	2	Legal, Social and Media analysis
Karsan *L	Aly	UBC	Co-App. Leader Vancouver site, BC lab testing	4	UBC MolDx Lab head, NGS expert, Vancouver site lead
Rouleau *L	Guy	UdMontréal	Co-App. Leader Mtl site, Ste-Justine lab testing	4	Ste-Justine MolDx Lab head, NGS expert, Mtl site lead.
Laberge *G	Anne-Marie	UdMontréal	Co-Lead Act4. ELSI issues, public health and KT	4	Public health genomics, prenatal testing, geneticist, ELSI and KT
Audibert *O	François	UdMontréal	Co-App. Montréal recruitment, analysis	2	Ob&Gyn, prenatal diagnosis expert

[2] Role includes: Project Leader, Co-Project Leader, Co-Applicant, End-User, Collaborator etc. Definitions of participant categories are provided in the *Guidelines for Funding Research Projects*.

GenomeCanada GenomeQuébec

Michaud *G	Jacques	UdMontréal	Co-App.	2	Ste-Justine Genetics and genomics, MolDx Lab, NGS expert
Giguère *H	Anik	U Laval	Co-App	2	KT expert
Wilson *O	Douglas	Alberta Health Services	Co-App. End-User	2	ObGyn, prenatal screening
Giguère *L	Yves	U.Laval	Co-App. Québec lab testing	4	MolDx, Genetic epidemiology, population screening
Forest *DL	Jean-Claude	U.Laval	Co-App. Study design and analysis	4	Chairs prenatal testing committee (PQ), T21 screening expert
Little *E	Julian	U Ottawa	Co-App. Act. 1 and 2	2	Clin epidemiology, prenatal and newborn surveillance; HTA
Luo *L	Wei	PHAC	Co-App. End-User	2	Manager of CCASS maternal, fetal and infant health surveillance
Gagné	Christian	U Laval	Co-App. Act 2	2	Computer engineer, AI expert for C/E studies
DeGuise *D	Michèle	INESSS	End-User Act. 1,2,3,4	1	INESSS Scientific Director
Lévesque *O	Isabelle	CHU de Qc	Collab. Act. 4	2	Head, Dept of Ob&Gyn. CHU de Qc
Vermeesch *L	Joris	UZ Leuven (BE)	Collaborator Act 1	1	Chair Dept. of Human Genetics; Coordinator Genomics core; Lab of Cytogenetics and Genome Research
Bekker	Hilary	Leeds U. (UK)	Collaborator Act 4	1	SDM expert
